# Utilization of 18s ribosomal RNA LAMP for detecting *Plasmodium falciparum* in microscopy and rapid diagnostic test negative patients

**DOI:** 10.1371/journal.pone.0275052

**Published:** 2022-10-06

**Authors:** Enoch Aninagyei, Adjoa Agyemang Boakye, Clement Okraku Tettey, Kofi Adjei Ntiri, Samuel Ohene Ofori, Comfort Dede Tetteh, Thelma Teley Aphour, Tanko Rufai

**Affiliations:** 1 Department of Biomedical Sciences, School of Basic and Biomedical Sciences, University of Health and Allied Sciences, Ho, Volta Region, Ghana; 2 Ghana Health Service, Greater Accra Region, Mayera-Faase Health Centre, Mayera-Faase, Ghana; 3 Medical Laboratory Department, Accra Technical University, Accra, Ghana; 4 Ghana Health Service, Municipal Health Directorate, Ga West Municipal, Amasaman, Ghana; 5 Ghana Health Service, Greater Accra Region, Ablekuma North Health Directorate, Accra, Ghana; 6 Ghana Health Service, Eastern Region, New Juaben South Municipal Health Directorate, Koforidua, Ghana; 7 Department of Epidemiology and Biostatistics, Arnold School of Public Health, University of South Carolina, Columbia, SC, United States of America; National Institute of Research in Tribal Health, INDIA

## Abstract

In this study, *Plasmodium falciparum* was detected in patients that were declared negative for malaria microscopy and rapid diagnostic test kit (mRDT), using *Plasmodium* 18s rRNA loop-mediated isothermal amplification (LAMP) technique. The main aim of this study was to assess the usefulness of LAMP assay for detecting pre-clinical malaria, when microscopy and mRDT were less sensitive. DNA was obtained from 100 μL of whole blood using the boil and spin method. Subsequently, the *Plasmodium* 18s rRNA LAMP assay was performed to amplify the specific *Plasmodium* 18s rRNA gene. Microscopy and mRDT negative samples [697/2223 (31.2%)] were used for this study. Compared to frequencies obtained for the other demographic variables, most of the patients were < 6 years (37.7%), females (59.0%), peri-urban dwellers (39.0%) and patients that sought outpatient department services (39.3%). Overall, the prevalence of *Plasmodium* 18s rRNA was 17.5%. when stratified by study variables, *Plasmodium* 18s rRNA LAMP positivity was higher in patients over 30 years [58/122 (54.2%)], males [69/122 (56.5%)], rural dwellers [69/122 (56.5%)] and patients that sought OPD services [68/122 (55.7%)]. The risk of being infected with *Plasmodium* when routine tests were negative was higher in 15–30-year group (OR = 3.03, 95% CI: 1.6–5.8, p = 0.0007), patients > 30 years (OR = 15.2, 95% CI: 8.3–27.7, p<0.001), males (OR = 2.1, 95% CI: 1.4–3.2, p = 0.0002) and rural dwellers (OR = 2.2, 95% CI:1.4–3.6, p = 0.0009). However, risk was lower in post-natal children (OR = 0.3, 95% CI: 0.18–0.51, p<0.001). Majority (81.5%) of the infected patients presented with headache, herpes labialis, diarrhea and vomiting. We demonstrated the lack of sensitivities of microscopy and mRDT for one-time diagnosis of malaria. Therefore, it is essential to utilize a sensitive technique such as *Plasmodium* 18s rRNA LAMP to increase the detection rate of *Plasmodium* infection.

## Introduction

Sub-Saharan Africans are still at risk of malaria [1]. Because of that, prompt diagnosis of malaria is essential especially in children under 5 years and in pregnant women, who are at increased risk of the disease. Until malaria rapid diagnostic test (mRDT) kits were introduced in the early 1990s [2], microscopy was the mainstay. However, malaria microscopy has been found to be limited in its sensitivity to detect malaria parasites [3].

Malaria microscopy is reported to be less sensitive to parasitemia, less than 250 parasites per microliter of blood [4]. Additionally, long turnaround times, inadequate trained microscopists, lack of quality control programs, and standardized staining reagents are some of the demerits of the malaria microscopy technique [3–5]. MRDTs were manufactured to cirmumvernt the challenges of microcopy [6]. MRDTs detect various parasite antigens, namely, *Plasmodium* lactate dehydrogenase, *Plasmodium* histidine-rich proteins 2 (PfHRP2) and *Plasmodium* aldolase [7]. However, kits based on PfHRP2 are very common [8]. That nothswithstanding, PfHRP2 based kits are being rendered useless due to the increasing frequency of *Plasmodium falciparum* with mutations that make them incapable to produce the HRP 2 protein, the main marker the majority brands of mRDTs are manufactured to detect [6, 9, 10]. In Ghana [11–13] and elsewhere [11, 14, 15], various frequencies of *P*. *falciparum* with HRP2 gene deletions have been recorded, which result in false negative mRDT testing based on HRP2.

To overcome these challenges associated with malaria diagnosis, molecular testing based on nucleic acid amplification technology (NAAT) has been evaluated in several studies. Conventional polymerase chain reaction (PCR) has been the most evaluated nucleic acid amplification based technique for the detection of the *P*. *falciparum* parasite [12, 13]. In recent times, variants of PCR have been used to detect the parasite [16]. Notwithstanding the foregoing, the PCR technology is sophisticated, providing results with delay, reserved for specialized laboratories, requires skilled labor, and reactions are prone to contamination [17]. To surmount the demerits of PCR and its variants, a novel NAAT, called loop-mediated isothermal amplification (LAMP) was developed in the year 2000 by Notomi and his colleagues [18].

Since then, the LAMP assay has been used to identify several gene targets. In a literature review by Wong et al. (2018), hundreds of microorganisms have been detected by the LAMP assay [19]. LAMP assay is gaining prominence due to its rapidity, user-friendliness, high sensitivity and specificity, stability of the *Bst* DNA polymerase to PCR inhibitors, and amenability of the technique to outside laboratory conditions [16–20]. Additionally, reaction outcomes can be judged visually and the use of simple heating devices such as water baths and heating blocks have been employed to reduce the testing costs [21–23]. In Ghana, the LAMP assay has been widely used to detect several pathogens, namely, *P*. *falciparum* [24], Mycobacterium tuberculosis [25] and M. ulcerans [26].

In this study, *P*. *falciparum* was detected in patients that were declared negative for malaria microscopy and mRDT, using *Plasmodium* 18s rRNA LAMP. This study is essential because a previous study detected malaria parasites in about 12% of patients that were initially tested negative for malaria with repeated sampling [14]. Therefore, early detection of the *Plasmodium* parasite in patients presenting with classical signs of malaria at baseline is essential for prompt management.

## Materials and methods

### Study design

This study was a cross-sectional study designed to detect *P*. *falciparum* in patients previously tested negative for malaria by microscopy and mRDT.

### Study sites, study population, and study duration

This study was carried out in Koforidua Polyclinic, a public health facility in the New Juaben South Municipality in the Eastern Region of Ghana. The facility is the first point of contact in the municipality for primary health care needs. The facility operates an outpatient department, laboratory unit, 24-hours detention units, antenatal services, labour and recovery wards, Eye unit, Ear, Nose and Throat unit and other essential service deliver points. Patients included in this study were those tested negative for malaria by both microscopy and mRDT. Microscopy and mRDT were done by Ghana Health Service certified malaria microscopists and trained biomedical scientist certified to perform mRDT through the On-site Training and Supportive Supervision (OTSS) programs. Samples were collected for this study from February—December 2019. Individuals recruited for this study were systematically selected, with prior written consent. Further, written consent was sought from parents or guardians of the minors prior to enrolling into this study.

### Inclusion and exclusion criteria

Patients included in this study were suspected of malaria but was initially tested negative for malaria parasites using microscopy and mRDT. To be included in the study, one or more of these clinical histories must be confirmed to be present by the attending clinicians, history of vomiting, presence of fever, chills, diarrhea, anemia, skin rashes, headache, nausea, mouth and lip sores, hematuria, and dizziness. Patients previously treated for malaria (inquired on direct questioning) on review visits were excluded from this study.

### Clinical assessment

A pediatrician, an obstetric and gynecologist, and a physician specialist examined each patient for overt and covert signs of malaria.

### Laboratory procedures

#### Sample collection

At least 4 mL of whole blood was collected from each patient suspected of malaria. Within 24 hours of sample collection, malaria microscopy [27] and mRDT [15] were done as previously described. Malaria negative concordant samples were further tested for *Plasmodium* 18s rRNA gene.

### Extraction of Plasmodium DNA

Equal volume (100 μL) of whole blood was mixed with an extraction buffer (400 mM NaCl, 40 mM Tris pH 6.5, 0.4% SDS). The mixture was briefly vortexed and subsequently boiled for 5 minutes at 95°C. The haemolysate was spun at 10,000 rpm for 3 minutes. The supernatant was frozen at -30°C before the LAMP assay.

### Detection of Plasmodium 18s rRNA gene by LAMP

The amplification of the *Plasmodium* 18s rRNA gene was done in pools of ten comprising 50μL of pooled DNA and master mix components made up of 1 μL, 2.6 μL, and 1 μL of fluorescent detector, primer mix, *Bst DNA polymerase*, respectively (Eiken Chemical, Japan) and 2.9 μL of nuclease free water in a total volume of 57.5 μL. Positive pools were tested individually using DNA volume of 5μL in a final volume of 12.5 μL using master mix components as stated above. This protocol has previously been published [24], using the LAMP primers presented in Table 1, which was initially used by [28].

**Table 1:** Oligonucleotide primers used for the 18s rRNA amplification.

Region	Oligonucleotide primers
FIP	5’-AGCTGGAATTACCGCGGCTGGGTTCCTAGAGAAACAATTGG-3’
BIP	5’-TGTTGCAGTTAAAACGTTCGTAGCCCAACCAGTTTAAATGAAAC-3’
F3	5’-TGTAATTGGAATGATAGGAATTTA-3’
B3	5’-GAAAACCTTATTTTGAACAAAGC-3’
LF	5’-GCACCAGACTTGCCCT-3’
LB	5’-TTGAATATTAAAGAA-3’

FIP–forward inner primer, BIP–backward inner primer, F3—forward outer primer, B3 –backward outer primer, LF—forward loop primer, LB—backward loop primer

### Amplification and visualization of 18s rRNA gene

**Fig 1:**
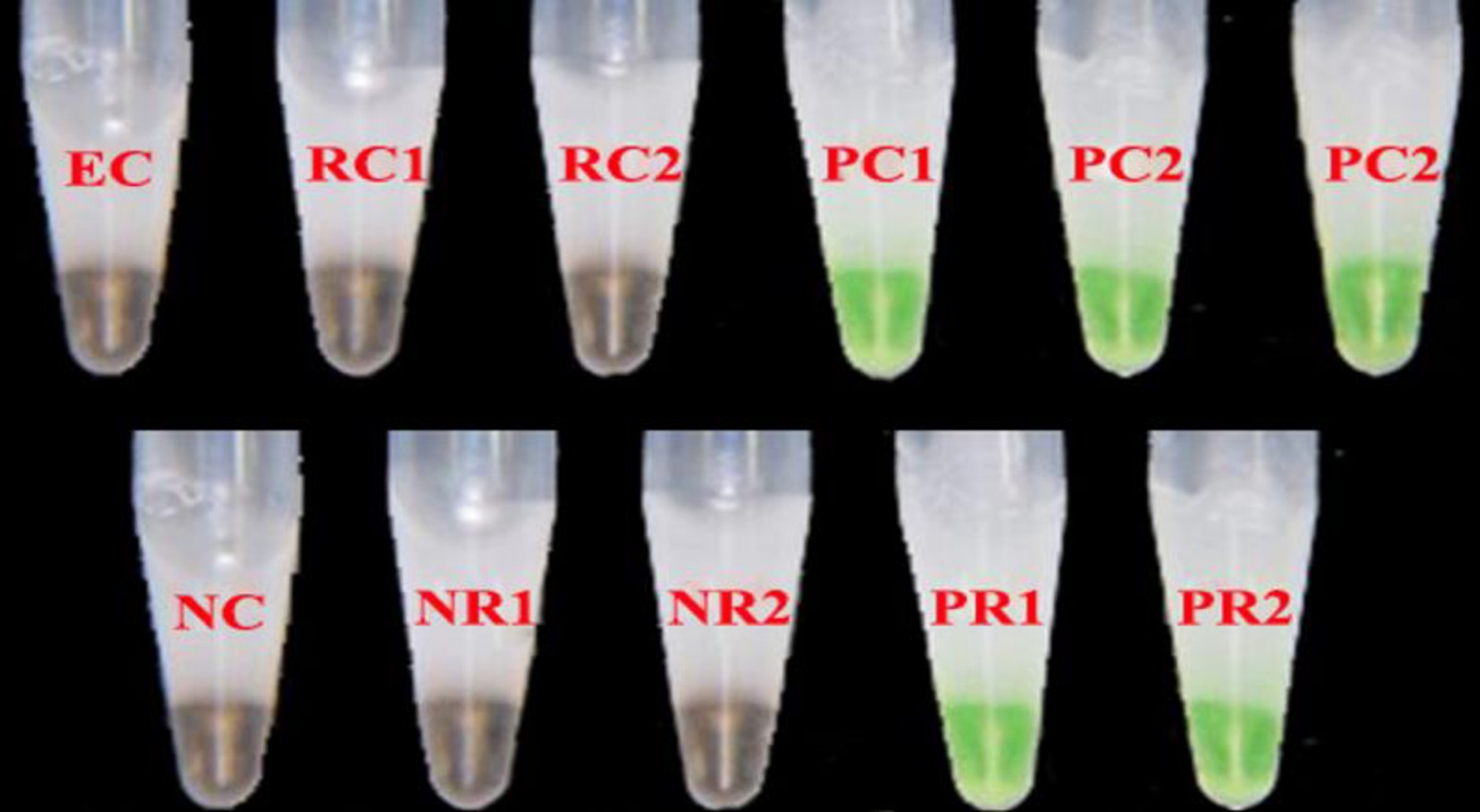
Quality control of LAMP assay. EC–extraction control, RC1 –reaction control (master mix), RC2—reaction control: master mix with nuclease free water, PC 1 –positive control (24,087 parasites/μL), PC2—positive control (812 parasites/μL), PC3—positive control (6 parasites/μL), NC–negative control, NR1/2 –patient negative reactions, PR1/2 –patient positive reactions.

The LAMP reaction was done using Stuart Heating Block (Cole-Parmer, Vernon, United States), at a constant temperature of 65°C for 60 minutes. The enzyme was inactivated at 80°C for 5 minutes. Per the fluorescent detector used, greenish yellow end product was corresponded with a positive reaction while the brownish product was a negative test (Fig 1).

### Internal quality controls

Six different controls were incorporated into the study, namely, positive controls (three blood samples with parasitemia, 24,087 parasites/μL, 812 parasites/μL and PBS diluted sample with estimated parasitemia 6 parasites/μL), extraction control (nuclease free water), and reaction controls (master mix). The extraction control, reaction control, and the master mix were *Plasmodium* LAMP negative while the positive controls were positive. The internal and patient control reactions are presented as Fig 1.

### Data analysis

Data analysis was done by using SPSS Version 25 (Chicago, IL, USA). Univariate analysis was made to determine the proportion *Plasmodium* 18s rRNA positivity rate in each demographic and clinical variable. This was presented as tables and graphs. Multivariate analysis was done to identify variables at risk of *Plasmodium* 18s rRNA infection. P-value less than 0.05 was considered statistically significant.

### Ethical approval

This study was approved by the Ghana Health Service Ethics Review Committee (GHS-REC002/03/18).

## Results

### Descriptive statistics of the outcome of the study samples

During the study period, 2223 patients suspected of malaria were recruited. Of this number, 579 (26%) and 947 (42.6%) patients were positive for microscopy and mRDT alone respectively wheras 568 (98.1%) samples out of the those positive for microscopy were positive for both micrscopy and mRDT. Of the total samples collected, 697 (31.4%) samples were negative for both microscopy and mRDT.

### Characteristics of the study participants and their association with *Plasmodium* 18s rRNA status

Table 2 presents the statistical analysis of the study data. Most of the patients were children up to five years (37.7%), females (59.0%), peri-urban dwellers (39.0%), and patients that sought outpatient department services (39.3%). Overall, the prevalence of *Plasmodium* 18s rRNA in patients with previous negative malaria test was 17.5%. Notwithstanding the foregoing, majority of *Plasmodium* 18s rRNA gene was detected in patients over 30 years [58/122 (54.2%)], males [69/122 (56.5%)], rural dwellers [69/122 (56.5%)] and patients seeking OPD services [68/122 (55.7%)]. The risk of having 18S LAMP positive result was increased in patients aged 15–30 years (OR = 3.03, 95%CI: 1.6–5.8, p = 0.0007) and over 30 years (OR = 15.2, 95%CI: 8.3–27.7, p < 0.001) compared to those aged less than 6 years. Furthermore, the risk of having 18S LAMP positive result was increased in patients in males (OR = 2.1, 95% CI: 1.4–3.2, p = 0.0002), rural dwellers (OR = 2.2, 95% CI:1.4–3.6, p = 0.0009) and decreased in peri-urban dwellers (OR = 0.4, 95% CI: 0.24–0.77, p = 0.005) relative to urban dwellers. Finally, patients that sought antenatal services (OR = 0.67, 95% CI: 0.41–1.08, p = 0.1), and child welfare clinic (OR = 0.3, 95% CI: 0.18–0.51, p<0.001) had a lower risk of *Plasmodium* 18s rRNA positivity compared to patient that sought outpatient services.

**Table 2:** Demographical features of the study participants and their association with *Plasmodium* 18s rRNA status.

		*Plasmodium* 18s rRNA			
Variable	Total	Negative n (%)	Positive n (%)	OR (95%CI)	p-value
Age range					
0–5	263 (37.7)	244 (92.8)	19 (7.2)	1	
6–14	202 (29.0)	181 (89.6)	21 (10.4)	1.5 (0.8–2.8)	0.229
15–30	125 (17.9)	101 (80.8)	24 (19.2)	3.03 (1.6–5.8)	0.0007
> 30	107 (15.4)	49 (45.8)	58 (54.2)	15.2 (8.3–27.7)	<0.001
Gender					
Male	286 (41.0)	217 (75.9)	69 (24.1)	2.1 (1.4–3.2)	0.0002
Female	411 (59.0)	358 (87.1)	53 (12.9)	1	
Residential setting					
Rural	229 (32.9)	160 (70.0)	69 (30.0)	2.2 (1.4–3.6)	0.0009
Peri-urban	271 (39.0)	250 (92.3)	21 (7.7)	0.4 (0.24–0.77)	0.005
Urban	197 (28.3)	165 (83.8)	32 (16.2)	1	
Service required					
Outpatient	274 (39.3)	206 (75.2)	68 (24.8)	1	
Antenatal	171 (24.5)	140 (81.9)	31 (18.1)	0.67 (0.41–1.08)	0.1
Child welfare clinic	252 (36.2)	229 (90.9)	23 (9.1)	0.3 (0.18–0.51)	<0.001

OR–odds ratio; P–value at 95% confidence interval

### Frequencies of *Plasmodium* 18s rRNA positivity in various rainfall patterns

**Fig 2:**
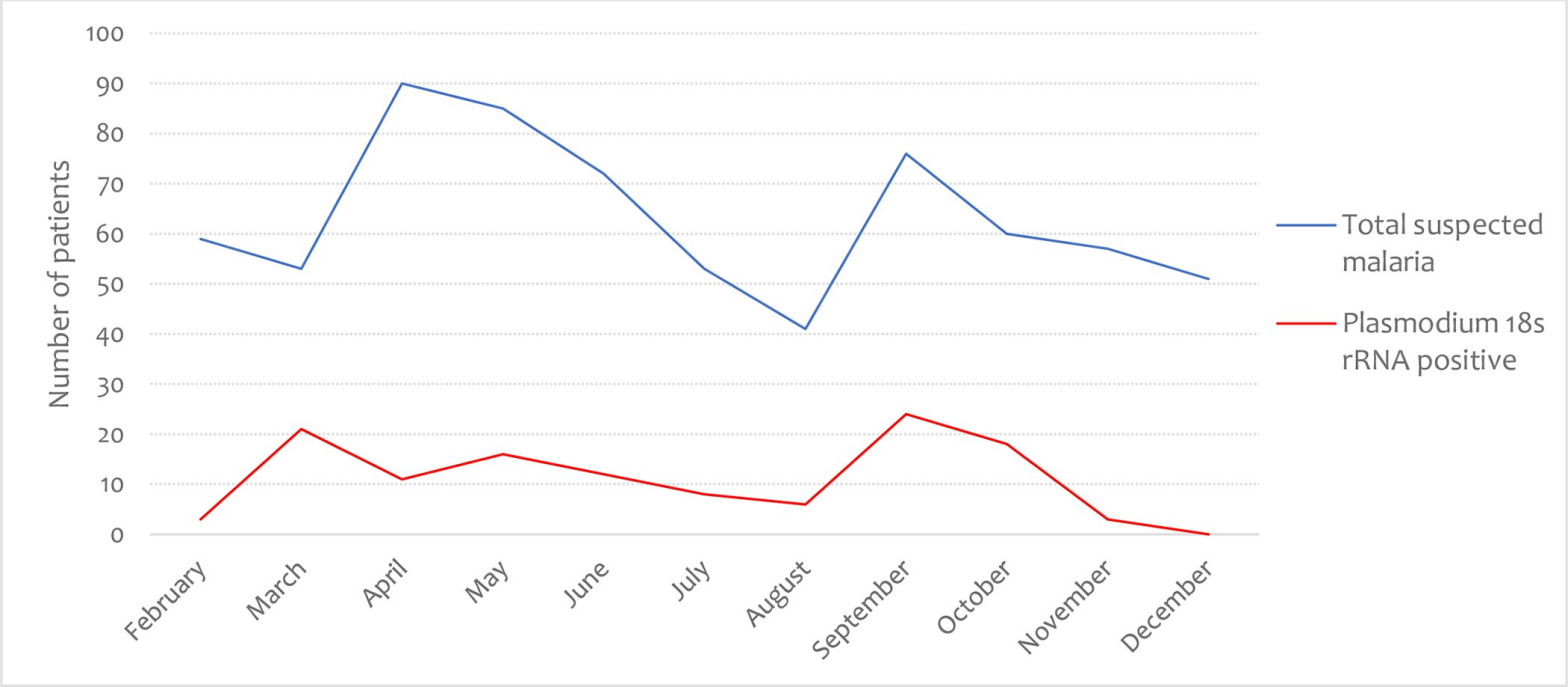
Monthly trends in suspected and *Plasmodium* 18s rRNA positive cases. Major rainfall–April–June, minor rainfall–March, September and October, no or very low rains—July, August, November–February.

Fig 2 details the monthly prevalence of subpatent malaria during the period of the study. The *Plasmodium* 18s rRNA prevailed during the season of minor rains [36.5% (63/189)] compared to the major rainy season [15.6% (39/247)] and the season of no or very low [7.7% (20/261)] rains. In the major rainy season, more cases of *Plasmodium* were observed in May [18.8% (16/85)] compared to June and April with 16.7%, and 12.2% respectably. During the season of minor rains, each month recorded 30% or more of *Plasmodium* 18s rRNA positivity, however, March recorded the highest positivity rate. In the season of no or very low rains, July, and August recorded 15.9% and 14.6% *Plasmodium* 18s rRNA positivity rate, respectively, while December recorded no positive Plasmodium 18r rRNA.

### Clinical history of patients infected with *P*. *falciparum*

Table 3 indicates the clinical history of the patients with *LAMP* positive reactions. The 122 patients found to be positive for 18S rRNA presented with a total of 190 symptoms. Majority of the patients (155/190; 81.5%) presented with headache (n = 50, 26.3%), herpes labialis (n = 39, 20.5%), diarrhea (n = 35, 18.4%), and vomiting (n = 31, 16.3).

**Table 3:** The proportions of the clinical features of the LAMP positive patients.

Clinical history	LAMP positive	%
**Headache**	50	26.3
**Herpes labialis**	39	20.5
**Diarrhea**	35	18.4
**Vomiting**	31	16.3
**Fever**	9	4.7
**Dizziness**	7	3.7
**Chills**	5	2.6
**Nausea**	5	2.6
**Skin rashes**	4	2.1
**Anemia**	3	1.6
**Hematuria**	2	1.1

## Discussion

This study reported that microscopy and mRDT underestimated the detection of *Plasmodium* in suspected patients by 17.5%. This could be due to the reported high sensitivity of LAMP for detecting malaria parasites, even in asymptomatic situations. In a previous study, the limit of detection for LAMP for detecting malaria parasites was 0.25 parasites/μL of blood [24]. In this study, the final product fluorescence observed for a sample dilution containing 6 parasites/μL of blood was similar to a sample containing 24,087 parasites/μL. this underscores the sensitivity of the LAMP assay, hence, able to detect *P*. *falciparum* is blood samples that previously was deemed negative by routine methods.

Children under 6 years and pregnant women were also found to be positive for 18s rRNA LAMP. These cohorts were mostly at risk of malaria, therefore, prompt treatment following early detection is essential to prevent deleterious sequelae associated with *Plasmodium* infection [22, 29]. The risk of submicroscopic malaria was significantly higher in patients 15 years or more compared to patients less than 6 years. The main reason could be due to competent *Plasmodium* immunity which increases with age [30]. This easily result in either asymptomatic or submicroscopic malara, therefore, children (especially under 6 years of age) easily developed clinical malaria compared to older patients due to incompetent antimalarial immunity [31]. It was also observed that the positivity rate was less in patients aged 6–29 years compared to patients over 30 years. It could possibly be that they were exposed to the parasite due to prolonged nocturnal and diurnal working life which is common among these age group [32].

Aninagyei (2020) reported that microscopy underestimated the detection rate of *Plasmodium falciparum* by 12% on single testing. When repeated testing was done at 6 hours and 12 hours after baseline testing, the positivity rates were 35% and 42%, respectively. This was because parasitemia at hour 6 was submicroscopic but at the 12^th^ hour, continuous and unchecked multiplication of the parasites yielded enough parasitemia to be detected microscopically [14]. During the same period, the positivity rate of mRDT increased from 42.9% at baseline testing to 47.5% 12 hours after baseline testing [14]. It is obvious that a significant proportion of patients presenting with clinical signs of malaria may test positive for malaria either with microscopy or mRDT within 12 hours or more. However, repeated sampling for 12 hours or more may not be feasible since those that temporarily responded to tepid sponging or other interventions may be discharged and may return to the hospital with a severe form of the disease. In the previous study the proportion malaria was underreported by 12% while in this study, LAMP was positive in only 7.2% that were previously tested negative by routine methods. This observation was quite surprising since a similar or higher prevalence was expected. The reason that could be ascribed to this observation was probably due to the differences in the prevalence of malaria in these study sites. The prevalence of malaria in the Ga West Municipality, a contiguous district to the study site for the Aninagyei (2020) study, was reported to be 36.3% [33], which was significantly higher than the prevalence of malaria in Kwahu-South (11.9%) [34], a contiguous district to the New Juaben South Municipality, the study district for this study. Secondly, the study site for the previous study was peri-urban while that of this study was urban. These factors could account for the differences of prevalence observed in this study.

The risk of *P*. *falciparum* positivity was reduced in children between 0–5 years and pregnant women. This could be as a result of the free distribution and use of long-lasting insecticide treated nets to children and pregnant women in Ghana [35] and the mandatory use of sulfadoxine/pyrimethamine prophylaxis by pregnant women which is directly administred by the health care providers in Ghana [36]. Additionally, *P*. *falciparum* cytoadherence especially in placenta [37] could account for the reduced sensitivity of the LAMP assay due possibly to very few presence of peripheral parasitemia.

Even though females have been found to be more likely to have malaria than males [38, 39], our study reported otherwise. The risk of 18s rRNA positivity was found to be more than doubled for male gender, Those studies reported on clinical malaria while in our study, subpatent malaria was the case. It was reported in another Ghanaian study [40] that being male increased the odds of asymptomatic malaria by 18%. The higher prevalence of asymptomatic malaria infections among males than females have been reported as a result of gender roles with regards to division of labour, hormonal or host genetic factors [41, 42].

On the other hand, rural dwellers have been reported in several studies to be associated with malaria transmission. This is because the vector for transmission of *Plasmodium* has been reported to survive well in forested areas where there is stagnation of clear rain water in crevices, humid atmosphere, and tree holes which favor oviposition sites. Additionally, the presence of temporary pools and gullies are common in rural areas [31, 32, 34, 35]. Furthermore, rural dwellers have the habit of outdoor activities, especially at night, when mosquito biting rates are high [43].

The study also found that *Plasmodium* infection was higher in the months of March, September, and October, compared to the other months. In these months, irregular rains were frequent. Irregular rainfall patterns offer enough time for the mosquito eggs to hatch into larvae and subsequently to the pupal stage and finally, to the adult stage. During these periods, room temperature rises due to frequent switches between sunny and short rains. Increased room temperature is associated with infrequent insecticide treated net usage [44]. Taking together, vector inoculation rates increase, which increase the carrier rate of the *Plasmodium* parasites. Unfortunately, there is no reported evidence of the actual state of entomological inoculation rates in the study municipality. There is the need to establish this, in a future study, to give credence to this observations.

## Conclusion

We demonstrate the lack of sensitivity of microscopy and mRDT for one-time diagnosis of malaria. These routine techniques have been shown to underestimate *Plasmodium* positivity rate by 17.5%, with a significant number of children (9.1%) and pregnant women (18.1%) declared negative for malaria. Therefore, it is essential to utilize a sensitive technique such as *Plasmodium* 18s rRNA LAMP to increase the detection rate of *Plasmodium* detection. In conclusion, LAMP technology is highly recommended for detection of subpatent parasitemia because it has been reported to be of high sensitivity, simple to use, cost-effective, and user-friendly [24].

## List of abbreviations

HRP: histidine-rich protein
LAMP: loop-mediated isothermal amplification
mRDT: malaria rapid diagnostic test kit
NAAT: nucleic acid amplification technologies
PCR: polymerase chain reaction
